# Outcomes in Patients With Multiple Myeloma and Prescribed Urate-Lowering Therapy: A Propensity-Matched Study

**DOI:** 10.7759/cureus.87183

**Published:** 2025-07-02

**Authors:** Sarah Eidbo, Maxim Barnett, Diego Lema Rodriguez, Nanuka Tsibadze, Alexander Pappas, Mohibur Shah, Abhishek Shah, Ryan Mayo

**Affiliations:** 1 Internal Medicine, Jefferson Einstein Philadelphia Hospital, Philadelphia, USA; 2 Internal Medicine, Einstein Healthcare Network, Philadelphia, USA; 3 Hematology and Oncology, Jefferson Einstein Philadelphia Hospital, Philadelphia, USA

**Keywords:** database, gout, multiple myeloma, trinetx, urate lowering therapy

## Abstract

Introduction

Multiple myeloma or MM is a plasma cell dyscrasia that causes monoclonal immunoglobulin accumulation and is associated with manifestations including hypercalcemia, anemia, bone pain, and renal dysfunction. Gout is also associated with electrolyte derangements that result in similar end-organ dysfunction. There is little information on outcomes in patients with both diagnoses.

Methods

Adult patients with MM were propensity-score matched according to age at index event, sex, demographics, and comorbidities. Patients were identified using the International Classification of Diseases (ICD)-10 codes through TriNetX’s US Collaborative Network and sorted into cohorts based on the prescription of urate-lowering therapies (ULTs). Three ULTs, allopurinol, febuxostat, and rasburicase, were studied individually. Cohorts of patients with MM and any ULT and those with MM but no ULT were also included. Outcomes were followed for five years to assess the rates of acute kidney injury (AKI), dialysis initiation (HD), seizures, atrial fibrillation/atrial flutter (AF/AFL), ventricular fibrillation/ventricular tachycardia (VF/VT), and all-cause mortality.

Results

When compared to patients with MM with any ULT, the cohort of patients with MM and no ULT demonstrated significantly lower rates of HD (HR 0.74, 95% CI 0.671-0.815, p<0.0001), AF/AFL (HR 0.99, 95% CI 0.924-1.061, p<0.0001), VF/VT (HR 0.853, 95% CI 0.752-0.968, p=0.0066), and mortality (HR 1.012, 95% CI 0.965-1.065, p<0.0001). The MM with no allopurinol group demonstrated significantly reduced rates of AKI (HR 0.901, 95% CI 0.839-0.968, p=0.0007), HD (HR 0.715, 95% CI 0.633-0.807, p<0.0001), seizures (HR 1.029, 95% CI 0.868-1.219, p=0.0012), AF/AFL (HR 1.006, 95% CI 0.921-1.098, p<0.0001), VF/VT (HR 0.837, 95% CI 0.718-0.975, p=0.0024), and mortality (HR 0.919, 95% CI 0.868-0.974, p<0.0001) compared to the MM with allopurinol cohort. The MM with no febuxostat cohort showed significantly reduced rates of AF/AFL (HR 1.009, 95% CI 0.762-1.335, p=0.004) compared to the MM cohort with febuxostat. The MM with no rasburicase cohort had significantly reduced risk of AF/AFL (HR 0.861, 95% CI 0.582-1.275, p=0.0381) compared to the MM with rasburicase cohort.

Conclusion

ULTs may increase the risk of requiring HD, developing cardiac arrhythmia, and potentially mortality in patients with MM. There appear to be some ULT-specific relationships that warrant further exploration. These findings could be related to the electrolyte disturbances and end-organ dysfunction of MM being compounded by those of gout. Further research should be conducted to elucidate any relationship between these diagnoses.

## Introduction

Multiple myeloma (MM) is a plasma cell hyperproliferative condition that can lead to anemia, bone pain and osteolytic lesions, renal dysfunction, and electrolyte derangements via monoclonal immunoglobulin overproduction, often resulting in end-organ damage [1-3]. Believed to start as a benign monoclonal gammopathy of undetermined significance (MGUS), MM is thought to arise from approximately 1% of MGUS cases per year [4,5]. The cause of this progression is unclear, though oncogenes including Neuroblastoma RAS viral oncogene homolog (NRAS), V-Raf murine sarcoma viral oncogene homolog B1 (BRAF), and V-Ki-ras2 Kirsten rat sarcoma viral oncogene homolog (KRAS) have been explored as possible causes [4-7]. Regardless of the cause, overproduction of immunoglobulins can induce a hyperviscous state that can lead to renal tubular damage alongside neurologic derangements [8]. Pancytopenia is often induced by overcrowding of the healthy bone marrow with clonal cell proliferation [8,9]. Bone marrow stroma involved in the proliferation is also theorized to over-express the receptor activator of nuclear factor kappa-B ligand (RANKL) protein, thus causing osteoclast activation followed by bone resorption [8,9]. This leads to some of the electrolyte derangements seen in MM [8,9]. 

MM is associated with electrolyte derangements including hypercalcemia, hypokalemia, hypophosphatemia, and hyperuricemia [8-12]. This is in part due to proximal renal tubular damage but also due to the rapid cell turnover occasionally seen in MM alongside the mechanisms discussed above [9-12]. Although patients with MM are not usually at a high risk of developing tumor lysis syndrome, it can occur in patients with high tumor burden or preexisting renal dysfunction due to the high purine metabolism from increased paraprotein synthesis [11,13]. In these patients, initiating urate-lowering therapies (ULTs) that are usually used in gout can be beneficial [11,13]. The most utilized ULTs include allopurinol, febuxostat, and rasburicase; each with their own indication [12-17]. Allopurinol, a xanthine oxidase inhibitor, is a standard prophylaxis in MM and is initiated before chemotherapy in those who are at risk of developing hyperuricemia [15]. Febuxostat is a selective xanthine oxidase inhibitor, but unlike allopurinol, it can be used in patients with renal dysfunction [16]. Rasburicase is a recombinant urate oxidase enzyme that is preferred for rapid urate lowering, such as in acute cases of tumor lysis syndrome [17]. 

These ULTs are indicated in patients with MM, independent of their gout diagnosis. However, patients with gout who are already prescribed ULTs may experience some protection against adverse effects if they are later diagnosed with MM as well. Although relationships appear to exist between the conditions, with gout being an occasional presenting feature of MM and patients with MM being at a higher risk of developing gout, there is little known about the conditions existing as comorbidities [18,19]. This study aimed to elucidate how a comorbid gout diagnosis would impact patients with MM. It also sought to determine how concomitant ULT use could affect outcomes for patients newly diagnosed with MM. 

## Materials and methods

Data extraction 

Deidentified health information was retrieved from the TriNetX US Collaborative network, a nationwide health information database that provides deidentified health information across multiple healthcare organizations (HCOs). Information was obtained by querying TriNetX according to International Classification of Diseases (ICD)-10 codes. Queried ICD-10 codes include those related to MM, gout, and the three ULTs: allopurinol, febuxostat, or rasburicase. These are included in Tables 1, 2, respectively.

**Table 1:** ICD-10 codes for multiple myeloma and gout International Classification of Diseases (ICD)-10 codes for multiple myeloma and gout. Data is condensed from the TriNetX output provided in the appendices.

Condition	ICD-10 code
Multiple Myeloma (MM)	UMLS:ICD10CM:C90.02: Multiple myeloma in relapse
	UMLS:ICD10CM:C90.0: Multiple myeloma
	UMLS:ICD10CM:C90.00: Multiple myeloma not having achieved remission
	UMLS:ICDO3:9732/3: Plasma cell myeloma
Gout	NLM:RXNORM:283821: Gout
	UMLS:ICD10CM:M10.9: Gout, unspecified

**Table 2:** ICD-10 codes for urate-lowering therapies International Classification of Diseases (ICD)-10 codes utilized for urate-lowering therapies (ULTs) studied. Data are condensed from the TriNetX output provided in the appendices.

Urate-lowering therapy	ICD-10 code
Allopurinol	NLM:RXNORM:519
Febuxostat	NLM:RXNORM:73689
Rasburicase	NLM:RXNORM:283821

ICD-10 codes were also utilized to identify outcomes including acute kidney injury (AKI), hemodialysis initiation (HD), seizures, atrial fibrillation or atrial flutter (AF/AFL), ventricular fibrillation or ventricular tachycardia (VF/VT), and all-cause mortality. The primary outcome studied was the all-cause mortality rate for each cohort, with the other listed outcomes considered as secondary outcomes. Outcome ICD-10 codes are listed in Table 3. 

**Table 3:** ICD-10 codes for outcomes ICD: International Classification of Diseases; AKI: Acute kidney injury or failure; HD: Initiation of dialysis; AF/AFL: Atrial fibrillation or atrial flutter; VF/VT: Ventricular fibrillation or ventricular tachycardia. Data are condensed from the TriNetX output provided in the appendices.

Outcome	ICD-10 Code
AKI	UMLS:ICD10CM:N17: Acute kidney failure
	UMLS:ICD10CM:N17.9: Acute kidney failure, unspecified
	UMLS:ICD10CM:N17.8: Other acute kidney failure
	UMLS:ICD10CM:N17.1: Acute kidney failure with acute cortical necrosis
	UMLS:ICD10CM:N17.0: Acute kidney failure with tubular necrosis
	UMLS:ICD10CM:N17.2: Acute kidney failure with medullary necrosis
	UMLS:ICD10CM:N17-N19: Acute kidney failure and chronic kidney disease
HD	UMLS:CPT:90945: Dialysis procedure other than hemodialysis (eg, peritoneal dialysis, hemofiltration, or other continuous renal replacement therapies), with single evaluation by a physician or other qualified health care professional
	UMLS:CPT:90947: Dialysis procedure other than hemodialysis (eg, peritoneal dialysis, hemofiltration, or other continuous renal replacement therapies) requiring repeated evaluations by a physician or other qualified health care professional, with or without substantial revision of dialysis prescription
	UMLS:SNOMED:108241001: Dialysis procedure
	UMLS:CPT:1006747: Hemodialysis Access, Intervascular Cannulation for Extracorporeal Circulation, or Shunt Insertion Procedures on Arteries and Veins
	UMLS:SNOMED:302497006: Hemodialysis
	UMLS:ICD9CM:39.95: Hemodialysis
	UMLS:CPT:1012752: Hemodialysis procedure
	UMLS:CPT:90935: Hemodialysis procedure with single evaluation by a physician or other qualified health care professional
	UMLS:CPT:1006747: Hemodialysis Access, Intervascular Cannulation for Extracorporeal Circulation, or Shunt Insertion Procedures on Arteries and Veins
	UMLS:CPT:90937: Hemodialysis procedure requiring repeated evaluation(s) with or without substantial revision of dialysis prescription
	UMLS:ICD10CM:Z99.2: Dependence on renal dialysis
Seizures	UMLS:ICD10CM:R56: Convulsions, not elsewhere classified
	UMLS:ICD10CM:G40.89: Other seizures
AF/AFL	UMLS:ICD10CM:I48.91: Unspecified atrial fibrillation
	UMLS:ICD10CM:I48.0: Paroxysmal atrial fibrillation
	UMLS:ICD10CM:I48.1: Persistent atrial fibrillation
	UMLS:ICD10CM:I48.2: Chronic atrial fibrillation
	UMLS:ICD10CM:I48.19: Other persistent atrial fibrillation
	UMLS:ICD10CM:I48.21: Permanent atrial fibrillation
	UMLS:ICD10CM:I48: Atrial fibrillation and flutter
	UMLS:ICD10CM:I48.20: Chronic atrial fibrillation, unspecified
	UMLS:ICD10CM:I48.11: Longstanding persistent atrial fibrillation
	UMLS:ICD10CM:I48.92: Unspecified atrial flutter
	UMLS:ICD10CM:I48.3: Typical atrial flutter
	UMLS:ICD10CM:I48.4: Atypical atrial flutter
	UMLS:ICD10CM:I48.9: Unspecified atrial fibrillation and atrial flutter
VF/VT	UMLS:ICD10CM:I49.01: Ventricular fibrillation
	UMLS:ICD10CM:I49.0: Ventricular fibrillation and flutter
	UMLS:ICD10CM:I47.2: Ventricular tachycardia
	UMLS:ICD10CM:I47.20: Ventricular tachycardia, unspecified
	UMLS:ICD10CM:I47.29: Other ventricular tachycardia
Mortality	Deceased: Deceased

Adults with a diagnosis of MM and gout were included in this study, and were sorted according to whether or not they were prescribed ULTs for comorbid gout. The day that the patient first met the criteria for a cohort was defined as the index event, with the criteria being diagnosis of MM with an additional prescription of ULT, either allopurinol, febuxostat, or rasburicase. Patients with any of the outcomes listed (Table 3) that occurred prior to the index event were excluded. Patients were followed for a time window of five years and outcomes during this period were included in the study. 

Patients were divided into cohorts according to the prescribed ULT. These included MM and gout without specified ULT, MM and allopurinol, MM and febuxostat, and MM and rasburicase. A cohort with MM and not on ULT was included as a control group. Propensity-matching was performed for each cohort prior to analyses, with cohorts matched for age at index event, sex, race, ethnicity, and comorbid conditions. The propensity-matching characteristics are listed in Table 4. 

**Table 4:** ICD-10 codes for propensity-score matching International Classification of Diseases (ICD)-10 codes used to identify demographics and conditions utilized for cohort propensity-score matching. Data are condensed from the TriNetX output provided in the appendices.

Demographic	ICD-10 Code
Age at index	AI
Female	F
Black or African American	2054-5
Male	M
White or Caucasian	2106-3
American Indian or Alaska Native	1002-5
Unknown race	UNK
Native Hawaiian or Other Pacific Islander	2076-8
Unknown ethnicity	UN
Not Hispanic or Latino	2186-5
Hispanic or Latino	2135-2
Other race	2131-1
Asian	2028-9
Neoplasms	C00-D49
Diseases of the respiratory system	J00-J99
Diseases of the nervous system	G00-G99
Diseases of the digestive system	K00-K95
Diseases of the circulatory system	I00-I99

Data analysis 

Survival analyses utilized in this study included Kaplan-Meier analyses with Log-Rank testing, HRs, and proportionality testing. Median survival and survival probability testing were also performed. A probability value, or p-value, of 0.05 or less was defined as statistically significant.

## Results

MM and any ULT 

Propensity-score matching revealed 11,631 patients in each cohort, diagnosed with MM with and without ULT. The mean age of the MM with no ULT cohort was 70.9±10.8 years at the index event, compared to 70.8±10.7 years in the MM with ULT cohort. The former group had 29.19% female patients (n=3,395) while the latter had 29.1% (n=3,850). The MM with no ULT cohort was made up of predominantly Caucasian patients (58.21%, n=6,770), followed by African American (23.56%, n=2,740), Asian (5.48%, n=637), and Hispanic/Latino participants (2.19%, n=255). Similarly, the MM with ULT cohort was mainly Caucasian (57.4%, n=6,676), followed by African American (23.38%, n=2,719), Asian (5.7%, n=663), and Hispanic/Latino (2.36%, n=275) participants. The MM with no ULT cohort demonstrated significantly reduced risk of HD, AF/AFL, VF/VT, and mortality compared to those with MM and ULT. However, there was no difference between the cohorts in terms of the rates of AKI or seizures (Table 5).

**Table 5:** Outcomes of patients with multiple myeloma (MM) with and without urate-lowering therapies (ULT) AKI: Acute kidney injury or failure; HD: Initiation of dialysis; AF/AFL: Atrial fibrillation or atrial flutter; VF/VT: Ventricular fibrillation or ventricular tachycardia. Outcomes after propensity-score matching for each comparison, MM without any ULTs or gout compared to MM and gout with each ULT as listed: allopurinol, febuxostat, and rasburicase. Statistical significance is defined as a p-value at or less than p=0.05. Cells with asterisks (*) indicate a reduced risk of this outcome in the cohort without ULT. Data displayed here are condensed from data provided in the appendices.

P-value	Group 1	Group 2	AKI	HD	Seizures	AF/AFL	VF/VT	Mortality
	MM no ULT	MM and gout	0.1728	0.0001*	0.2231	0.0001*	0.0066*	0.0001*
	MM no ULT	MM and allopurinol	0.0007*	0.0001*	0.0012*	0.0001*	0.0024*	0.0001*
	MM no ULT	MM and febuxostat	0.574	0.1755	0.4207	0.0004*	0.113	0.1718
	MM no ULT	MM and rasburicase	0.1117	0.7466	0.1814	0.0381*	0.3104	0.0851
HR	Group 1	Group 2	AKI	HD	Seizures	AF/AFL	VF/VT	Mortality
	MM no ULT	MM and gout	0.836	0.74*	0.853	0.99*	0.853*	1.012*
	MM no ULT	MM and allopurinol	0.901*	0.715*	1.029*	1.006*	0.837*	0.919*
	MM no ULT	MM and febuxostat	0.641	0.325	1.152	1.009*	0.708	0.874
	MM no ULT	MM and rasburicase	0.536	0.539	0.65	0.861*	1.08	0.382
95% CIs	Group 1	Group 2	AKI	HD	Seizures	AF/AFL	VF/VT	Mortality
	MM no ULT	MM and gout	0.793-0.882	0.671-0.815*	0.96-1.278	0.924-1.061*	0.752-0.968*	0.965-1.065*
	MM no ULT	MM and allopurinol	0.839-0.96*	0.633-0.807*	0.868-1.219*	0.921-1.09*	0.718-0.975*	0.868-0.974*
	MM no ULT	MM and febuxostat	0.494-0.832	0.228-0.462	0.637-2.081	0.762-1.335*	0.432-1.16	0.729-1.048
	MM no ULT	MM and rasburicase	0.341-0.843	0.347-0.838	0.333-1.268	0.582-1.275*	0.619-1.885	0.309-0.473

MM and allopurinol 

Propensity-score matching revealed 7,652 patients per cohort. In the MM with no allopurinol cohort, the average age at index event was 70.4±10.6 years, compared to 70.4±10.5 years in the MM with allopurinol cohort. The former cohort had 26.54% female patients (n=2,031), compared to 26.52% female members (n=2,029) in the latter. The MM with no allopurinol cohort had mostly Caucasian members (61.61%, n=4,714), followed by African American (25.22%, n=1,930), Asian (4.48%, n=343), and Hispanic/Latino (2.76%, n=211) participants. The MM with allopurinol cohort was similarly made up of predominantly Caucasian patients (61.23%, n=4,685), followed by African American (24.69%, n=1,889), Asian (4.55%, n=348), and Hispanic/Latino (2.98%, n=228) members. The MM with no allopurinol cohort demonstrated significantly reduced risk of AKI, HD, seizures, AF/AFL, VF/VT, and mortality compared to the MM with allopurinol cohort (Table 5).

MM and febuxostat 

After propensity-score matching, 768 patients were identified in each cohort: MM with and without febuxostat. The average age at index event was 71±11.3 years for the MM with no febuxostat cohort, compared to 70.5±11.2 years for the MM with febuxostat one. The former group comprised 29.3% female patients (n=225), compared to 29.0% (n=223) in the latter. The MM with no febuxostat cohort had predominantly Caucasian participants (42.58%, n=327), followed by Asian (24.09%, n=185), African American (16.54%, n=127), and Hispanic/Latino (2.21%, n=17) members. The MM with febuxostat cohort was similarly made up of mostly Caucasian patients (43.1%, n=331), followed by Asian (23.31%, n=179), African American (16.28%, n=125), and Hispanic/Latino (2.08%, n=16) participants. The MM with no febuxostat cohort demonstrated significantly reduced risk of AF/AFL compared to the MM with febuxostat cohort whereas there were no differences between them in the rates of AKI, HD, seizures, VF/VT, or mortality (Table 5).

MM and rasburicase

Propensity-score matching identified 433 patients per cohort for patients with MM with and without rasburicase. In patients with MM and no rasburicase, the average age at index event was 67±10.6 years, compared to 66.7±10.7 years in those with MM and rasburicase. The former group had 23.56% female patients (n=102), compared to 23.79% (n=103) in the latter. The MM with no rasburicase cohort was made up of predominantly Caucasian members (60.97%, n=264), followed by African American (22.63%, n=98), Asian (6.69%, n=29), and Hispanic/Latino (2.77%, n=12) participants. Similarly, the MM with rasburicase cohort consisted of mostly Caucasian participants (60.74%, n=263), followed by African American (22.17%, n=96), Asian (6.69%, n=29), and Hispanic/Latino (3.23%, n=14) patients. The MM with no rasburicase cohort demonstrated significantly decreased risk of AF/AFL compared to the MM with rasburicase cohort. There were no differences between them in the rates of AKI, HD, seizures, VF/VT, or mortality (Table 5).

## Discussion

This retrospective analysis of a nationwide database involved patients with MM and comorbid gout on one of three ULTs and compared them to patients with MM and no gout without ULTs. ULTs, typically indicated for patients with gout, can also be utilized in patients with MM and a significant disease burden or in those who are about to initiate chemotherapy [11,13]. There is a paucity of data exploring the relationship between a concomitant diagnosis of both MM and gout. As such, limited data are available to characterize how ULT continuation, rather than initiation, could impact outcomes in MM. To best determine the relationship between gout, MM, and ULTs, cohorts of patients with MM and gout on one of three ULTs were compared with propensity-score matched cohorts of patients with MM and without gout, and not on ULTs. Outcomes were further studied for MM with comorbid gout and any ULT compared to MM without gout or any ULT. 

When cohorts were compared for the diagnosis of gout, our data revealed a significantly reduced risk of requiring HD, developing AF/AFL, developing VF/VT, and all-cause mortality in patients with MM and without gout or ULTs. Independently, chronic unmanaged gout can lead to urate nephropathy that can cause tubulointerstitial damage, with an eventual decline in renal function to the point of requiring HD initiation [20]. It could follow that with the additional burden of MM, the synergistic effect was too much to overcome. The electrolyte derangements seen in gout alone have demonstrated an increased risk for cardiac arrhythmias, most commonly AF but also AFL, VF, and VT [21]. This corresponds to the significant differences in the risk for AF/AFL and VF/VT seen in this study. It is likely that the additional electrolyte derangements conferred by MM compound those in gout, leading to cardiac arrhythmias. This significant difference in the risk of AF/AFL is seen across all ULT comparisons, with cohorts on any ULT being at a significantly higher risk of developing them. Given that AF is the arrhythmia most commonly associated with gout, this association is in line with current research [21]. The significant difference in all-cause mortality is another finding that can be explained in part by the findings discussed above. As the cardiac arrhythmias discussed can be potentially fatal, this could contribute to the significant risk difference in mortality seen between cohorts. However, these findings should be further explored, and other possible contributing factors better characterized. 

Interestingly, all the outcomes demonstrated a significant risk difference between cohorts with and without allopurinol. As allopurinol is frequently prescribed in patients with MM to prevent secondary hyperuricemia, it is interesting that patients already on the medication at the time of MM diagnosis demonstrated a higher risk of these outcomes. Despite allopurinol’s urate-lowering effects, our data suggest that comorbid gout may confer an additional risk that is not adequately controlled by the drug. 

Aside from the significantly increased rates of AF/AFL discussed above, cohorts comparing both febuxostat and rasburicase to cohorts without comorbid gout demonstrated no significant differences in outcomes. It is possible that this is due to the smaller group sizes, with febuxostat and rasburicase cohorts at 768 and 433 patients each. Of note, febuxostat and rasburicase are less commonly prescribed ULTs in gout, but with some key differences. Febuxostat operates by a nearly identical mechanism to allopurinol but does not require dose adjustment in patients with mild-to-moderate renal impairment [22]. As renal impairment interferes with allopurinol dosing, this is another possible variable at play in the significant risk difference between the cohorts with and without allopurinol. With febuxostat, however, multiple trials and meta-analyses comparing it with allopurinol have demonstrated non-inferior urate-lowering effects with similar safety profiles but reduced hypersensitivity reactions [22,23]. Further research should focus on exploring these differences between allopurinol and febuxostat. 

Rasburicase, a first-line ULT for patients with MM requiring rapid urate reduction, also did not change rates of outcomes significantly [17]. The only studied outcome that differed significantly was the rate of new AF/AFL development, the risk for which was higher in patients with MM on rasburicase. This study observed outcomes among patients who were prescribed rasburicase at the time of the index event, i.e. diagnosis of MM. Further research should explore how the outcomes are affected when rasburicase is prescribed after the diagnosis. 

Limitations

This study has several limiting factors that warrant discussion. Reliance on the TriNetX queries of ICD-10 coding is a potential source of error, particularly if patients are inaccurately coded. Inaccurate coding can lead to incorrect inclusion or exclusion of patients from queries and thus from cohorts. As TriNetX also relies on health care organizations (HCOs) to provide data during these queries, HCOs serve as an additional source of potential errors. HCOs may not respond to queries for a variety of reasons. Any omitted HCO data could have altered the results of this study in ways that are difficult to assess. Propensity-score matching further serves as a potential source of error. Though confounding variables are accounted for as thoroughly as possible via this method, it is always possible that this matching process was inadequate. Given that this study utilized a retrospective design, no causal relationships can be identified, and our data should be interpreted with caution and within the appropriate context. 

A limitation unique to this study is an inability to stratify patients based on malignancy burden or serum urate levels at the time of indexing. As the ULTs studied are indicated in patients with elevated serum urate levels, it would be interesting to see how these levels would impact outcomes in each cohort. It is possible that cohorts with preexisting gout and already initiated on ULTs would present with lower serum urate levels and possibly have better overall outcomes, though this cannot be stated without further research. The characterization of the impact of gout on MM outcomes should be further explored. 

## Conclusions

In conclusion, our data suggest that ULTs may increase the risk of several outcomes studied here, including the risk of requiring HD, developing cardiac arrhythmias, and even potentially mortality in patients with comorbid MM. There appear to be potential ULT-specific relationships that should be further explored, but may be attributable to differences in indication or mechanism of action. These findings may be related to the electrolyte disturbances and end-organ dysfunction in MM being compounded by those seen in gout. Further research is necessary to determine possible relationships between these diagnoses. 

## Figures and Tables

**Figure 1:**
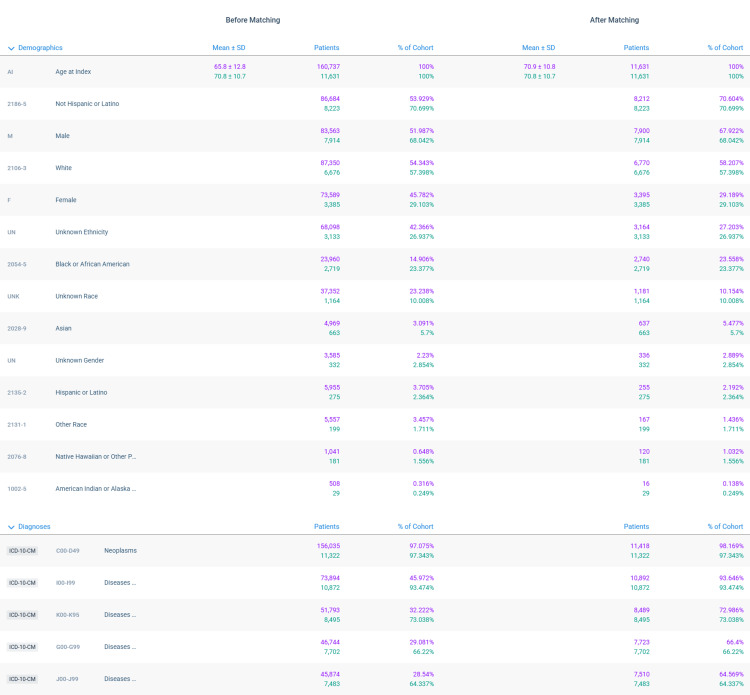
Patient characteristics at baseline for the cohorts with multiple myeloma (MM) with and without any urate-lowering therapy (ULT)

**Figure 2:**
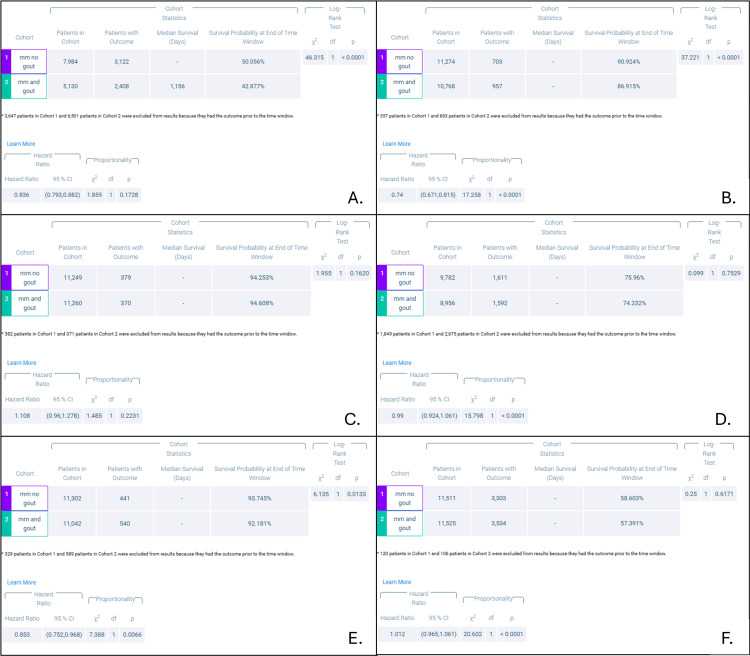
Differences in various outcomes between cohorts of multiple myeloma (MM) with and without any urate-lowering therapy (ULT) Panel A: Acute kidney injury (AKI); Panel B: Hemodialysis initiation (HD); Panel C: Seizures; Panel D: Atrial fibrillation or atrial flutter (AF/AFL); Panel E: Ventricular fibrillation or ventricular tachycardia (VF/VT); and Panel F: All-cause mortality.

**Figure 3:**
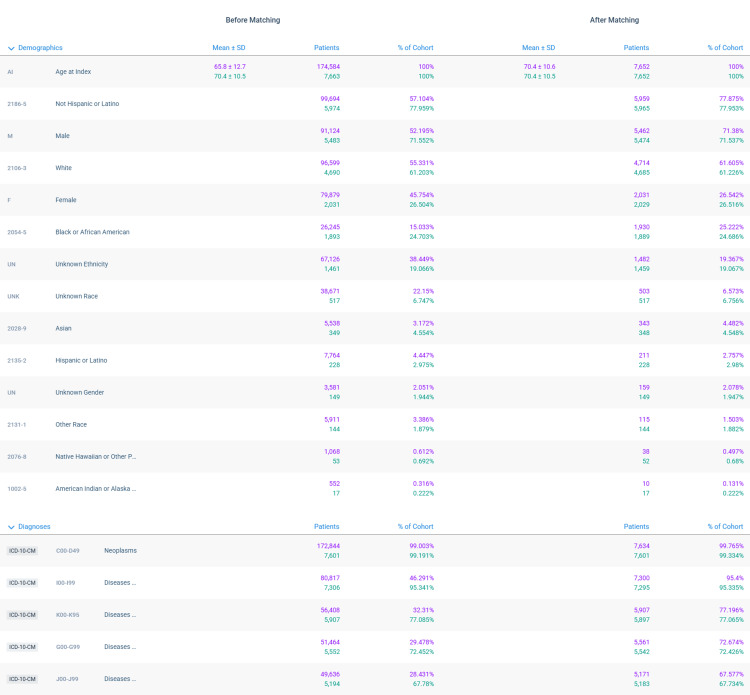
Patient characteristics at baseline for the cohorts with multiple myeloma (MM) with and without allopurinol

**Figure 4:**
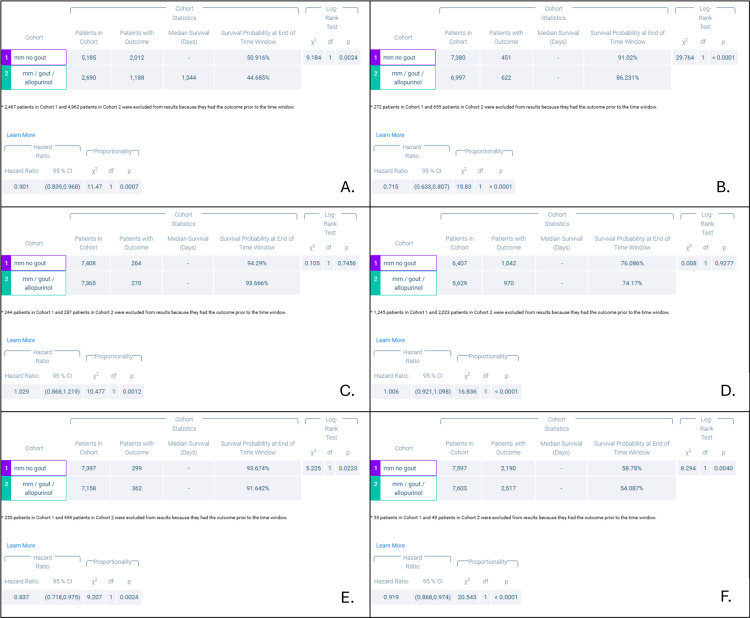
Differences in various outcomes between cohorts of multiple myeloma (MM) with and without allopurinol Panel A: Acute kidney injury (AKI); Panel B: Hemodialysis initiation (HD); Panel C: Seizures; Panel D: Atrial fibrillation or atrial flutter (AF/AFL); Panel E: Ventricular fibrillation or ventricular tachycardia (VF/VT); and Panel F: All-cause mortality.

**Figure 5:**
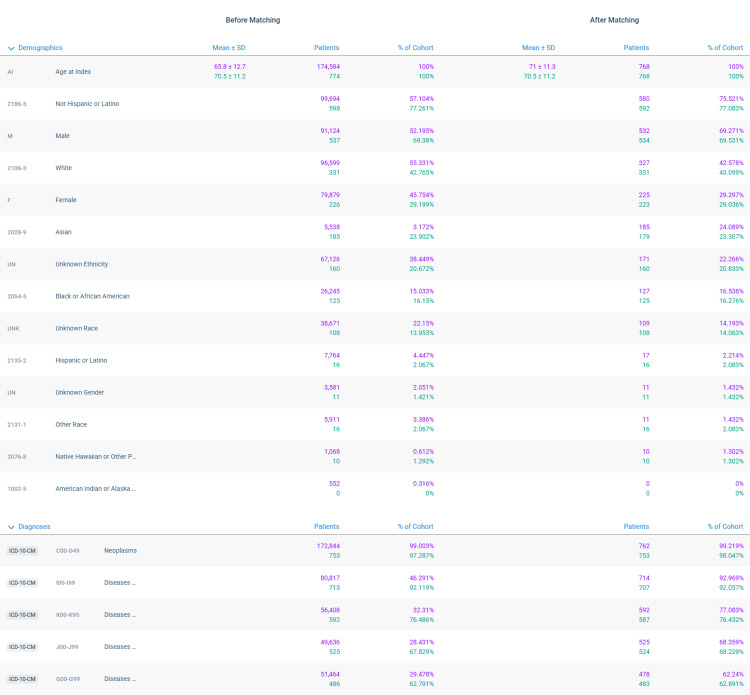
Patient characteristics at baseline for the cohorts with multiple myeloma (MM) with and without febuxostat

**Figure 6:**
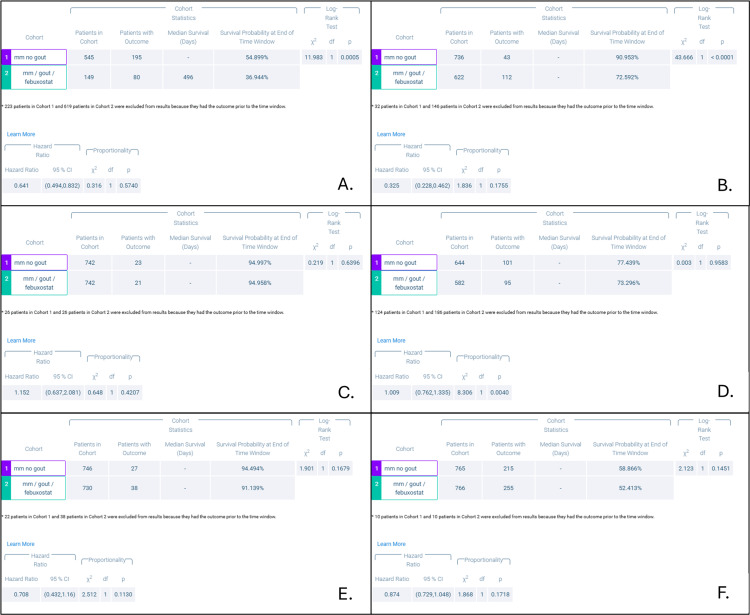
Differences in various outcomes between cohorts of multiple myeloma (MM) with and without febuxostat Panel A: Acute kidney injury (AKI); Panel B: Hemodialysis initiation (HD); Panel C: Seizures; Panel D: Atrial fibrillation or atrial flutter (AF/AFL); Panel E: Ventricular fibrillation or ventricular tachycardia (VF/VT); and Panel F: All-cause mortality.

**Figure 7:**
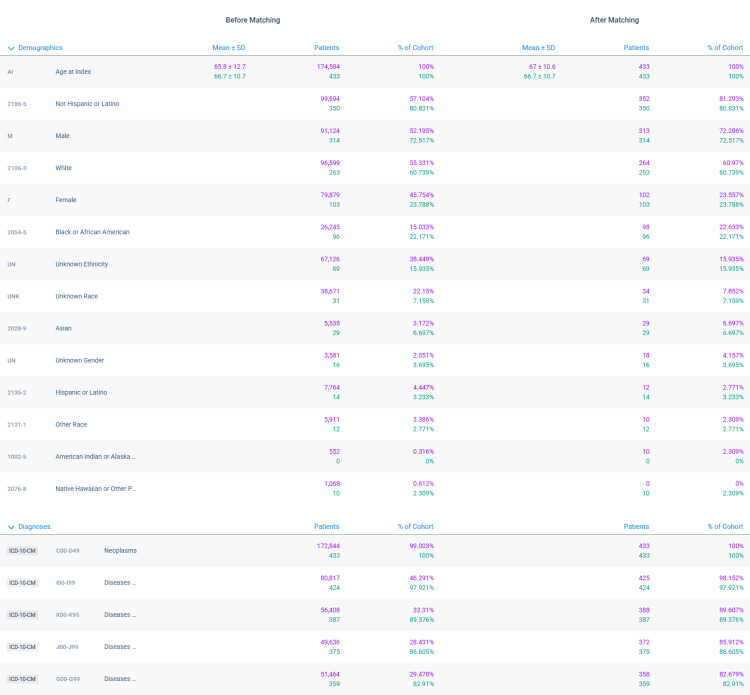
Patient characteristics at baseline for the cohorts with multiple myeloma (MM) with and without rasburicase

**Figure 8:**
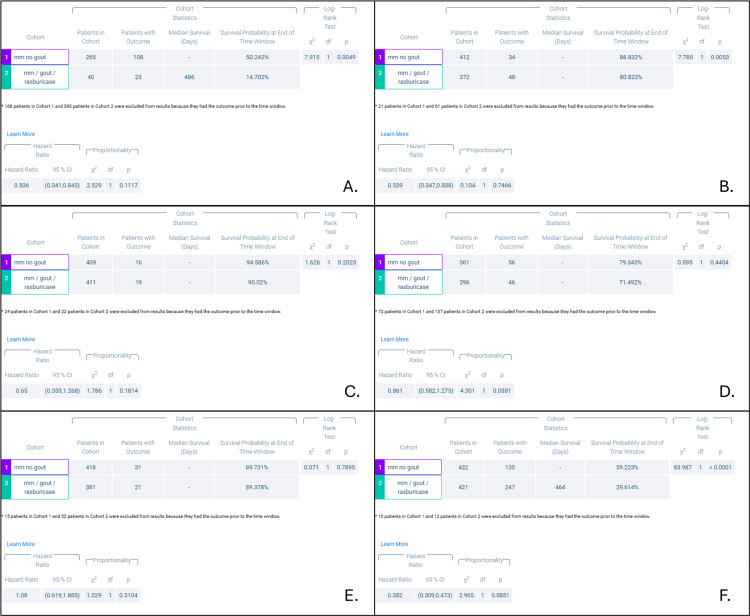
Differences in various outcomes between cohorts of multiple myeloma (MM) with and without rasburicase Panel A: Acute kidney injury (AKI); Panel B: Hemodialysis initiation (HD); Panel C: Seizures; Panel D: Atrial fibrillation or atrial flutter (AF/AFL); Panel E: Ventricular fibrillation or ventricular tachycardia (VF/VT); and Panel F: All-cause mortality.

## Appendices

MM and any ULT

Figures here correspond to the TriNetX query output obtained after comparing cohorts with MM with and without any form of ULT.

Figure 1 refers to the baseline characteristics of each cohort, before and after propensity-score matching for these characteristics and according to the ICD-10 codes listed at the bottom of the figure. 

Figure 2 displays the HR, 95% CI, and the p-value of the comparison of MM with and without any ULT for rates of AKI, HD, seizures, AF/AFL, VF/VT, and all-cause mortality. 

MM and allopurinol

Figures here correspond to the TriNetX query output obtained after comparing cohorts with MM with and without allopurinol.

Figure 3 refers to the baseline characteristics of each cohort, before and after propensity-score matching for these characteristics and according to the ICD-10 codes listed at the bottom of the figure. 

Figure 4 displays the HR, 95% CI, and the p-value of the comparison of MM with and without allopurinol for rates of AKI, HD, seizures, AF/AFL, VF/VT, and all-cause mortality. 

MM and febuxostat

Figures here correspond to the TriNetX query output obtained after comparing cohorts with MM with and without febuxostat.

Figure 5 refers to the baseline characteristics of each cohort, before and after propensity-score matching for these characteristics and according to the ICD-10 codes listed at the bottom of the figure. 

Figure 6 displays the HR, 95% CI, and the p-value of the comparison of MM with and without febuxostat for rates of AKI, HD, seizures, AF/AFL, VF/VT, and all-cause mortality.

MM and rasburicase

Figures here correspond to the TriNetX query output obtained after comparing cohorts with MM with and without rasburicase.

Figure 7 refers to the baseline characteristics of each cohort, before and after propensity-score matching for these characteristics and according to the ICD-10 codes listed at the bottom of the figure. 

Figure 8 displays the HR, 95% CI, and the p-value of the comparison of MM with and without rasburicase for rates of AKI, HD, seizures, AF/AFL, VF/VT, and all-cause mortality.  
